# Injury incidence in elite youth field hockey players at the 2016 European Championships

**DOI:** 10.1371/journal.pone.0201834

**Published:** 2018-08-23

**Authors:** Laura-Anne M. Furlong, Udo Rolle

**Affiliations:** 1 School of Sport, Exercise and Health Sciences, Loughborough University, Loughborough, Leicestershire, United Kingdom; 2 National Centre for Sport and Exercise Medicine–East Midlands, Loughborough, Leicestershire, United Kingdom; 3 Department of Pediatric Surgery and Pediatric Urology, University Hospital Frankfurt, Theodor-Stern-Kai 7, Frankfurt/M, Germany; 4 Fédération Internationale de Hockey (FIH) Health and Safety Panel, Lausanne, Switzerland; University of Victoria, CANADA

## Abstract

Despite being an essential consideration when deciding rule changes, injury prevention strategies, and athlete development models, there is little epidemiological data of U18 field hockey player injuries–something explicitly referred to in the 2015 International Olympic Committee’s Consensus Statement on Youth Athlete Development. The aim of this study was to quantify incidence and characteristics of injuries in elite youth field hockey players during a major international tournament. Standardized reporting forms detailing time, location on pitch, mechanism and anatomical location of injury were completed for new musculoskeletal conditions resulting in a time stoppage by the umpire and where a player was noticeably affected by an injury for up to 20 s regardless of time stoppage. Injury incidence was 1.35 and 2.20 injuries/match or 53 and 86 injuries per 1000 player match hours for boys (B) and girls (G) respectively; girls were over three times more likely to have a minor injury. Most injuries were contusions due to being hit by the ball or stick (B: 12, G: 27), with high numbers of injuries to the torso (B: 8) and head/face (G: 7). Injuries during the penalty corner (B: 3, G: 4) were to the lower limb and hand, and boys were less likely to wear facial protection (B: 65.9%, G: 86.4%). Results form an essential initial dataset of injuries in U18 field hockey players. Current reporting protocols under-report injuries and must be addressed by the international governing body. The high number of head/face injuries, particularly in females, requires further investigation.

## Introduction

The International Olympic Committee’s consensus statement on youth athlete development explicitly states the importance of adopting an evidence-based, holistic approach when working with young sportspeople to maximize enjoyment and involvement in physical activity [1]. A key consideration in developing technical and tactical proficiency within a sport is ensuring it occurs in as safe an environment as possible. Implementation of successful injury prevention strategies within youth sport is multi-factorial, but an essential first step requires establishing the extent of the injury problem [2, 3]. Understanding the nature and severity of all injuries incurred in a sport and the strategies used by athletes to minimize these risks are the underpinning of both injury prevention and athlete development models [4].

There is, in general, significantly less epidemiological data related to injuries in elite youth athletes compared to adults [5], and the 2015 consensus statement specifically refers to this in relation to field hockey [1]. The general paucity of field hockey injury literature has been highlighted in the recent systematic review by Delfino Barboza et al. [6]. Since 1975, eight full body injury datasets from school-aged field hockey players have been reported [7–15], as the Hootman et al. [14] and Dick et al. [13] papers report data from the same pool of players. Despite significant changes in the nature of the sport of field hockey in recent times (e.g. introduction of the autopass, allowing players to wear facemasks during penalty corners), only five papers reporting injury incidence have been published in the last ten years [15–19], of which only one has focused on developmental athletes aged, on average, 18 years [15]. None focus on the developing youth (U18) athlete. This distinct lack of evidence makes establishing the scale of the injury problem in youth field hockey practically impossible and limits the ability of practitioners and physicians to implement evidence-based injury prevention strategies in the sport.

Multi-day tournaments have an important role to play in talent identification, player and squad development in underage elite field hockey. There is a known increased risk of injury in competition settings, compared to training, in team sports such as soccer [20]. This risk increases further when high volume of matches are played in a short space of time, e.g. with fixture congestion [21] or at tournaments, due to potential cumulative fatigue. There are also known differences in injury risks between youth and adult athletes [22]. Theilen et al. [18] reported an injury incidence rate of 1.2 and 0.7 injuries per match for senior male and female hockey players at major tournaments, with the majority of injuries found to be contusions as a result of being struck with the ball or stick. It is unclear if the injury incidence and nature of injuries incurred with younger age groups is similar, due to different technical abilities and different team preparation strategies prior to competition. To the author’s knowledge, there is no existing dataset of injuries in U18 field hockey players in a modern tournament setting, despite their important role in athlete development.

As the governing body of hockey across the five continents, the International Hockey Federation (Fédération Internationale de Hockey, FIH) is responsible for deciding the official rules of the sport, permissible playing equipment, and establishing guidelines for optimal player management at all levels of the sport. Current competition reporting protocols require completion of an injury report form when any new musculoskeletal condition occurs on the pitch requiring a time stoppage. The existing protocol captures serious injuries but misses more minor injuries which do not lead to time stoppage but still lead to a player being negatively affected. There is hence a need to establish an evidence base of all injuries that occur with youth athletes, to develop both injury prevention strategies and athlete development models for the sport.

Despite the high number of youth field hockey players worldwide, there is a paucity of data related to the nature and severity of injuries in this population and the strategies used by players to minimize injury risk. This significantly limits potential to implement effective injury prevention strategies and optimize long-term development models. The aim of this study was to investigate injuries that occur in elite youth field hockey players at a major tournament using an adaptation to existing international governing body reporting protocols, and establish player use of protective equipment. These findings will provide the first insight into injuries in elite U18 hockey players in this setting. They will potentially identify what injuries treating clinicians and practitioners need to be aware of for tournament care, and how injury risk can be minimized in this group hence improving player safety.

## Methods

Following review and approval by the Loughborough University Ethics Approvals (Human Participants) Sub-Committee (approval number C16-39), all data was collected at the European U18 Hockey Championships I held in Cork, Ireland in July 2016. Sixteen national representative teams were in attendance, 8 in each of the boys’ and girls’ competitions. Injuries were analyzed for the boys’ and girls’ competitions separately.

**Fig 1:**
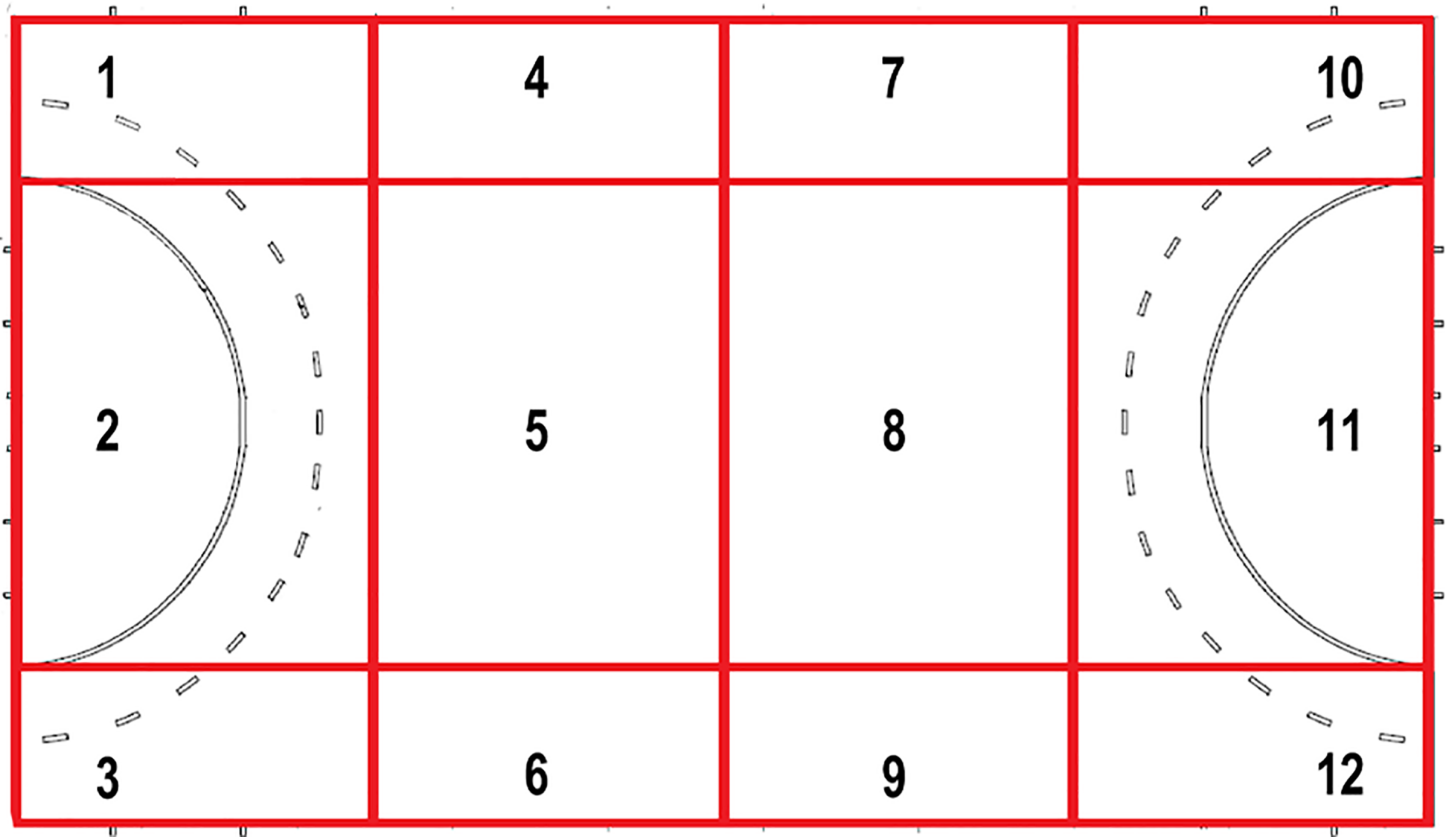
Coding used to identify where on pitch injuries occurred. Areas 1–3 and 10–12 were classed as inside the 23-meter lines, areas 4–9 were classed as between.

Current FIH medical reporting requires an official medical reporting sheet be completed if any new musculoskeletal complaint or concussion occurs which requires time to be stopped by the umpire. For the purpose of this study, these injuries were defined as serious injuries. As this may lead to underreporting of overall injuries, reporting sheets were also completed when the authors (n = 2) or officials (n = 12) noticed an obvious incident which affected a player’s ability to engage fully in the match for a brief period (up to 20 s) but was not serious enough to merit a time stoppage; for the purpose of this study, these injuries were classed as minor injuries. Official FIH reporting sheets require details on time in the match the injury occurred, team and player number (not included in this analysis but a standard part of the reporting form), area of body affected, mechanism and type of injury, and whether time was stopped by the umpires. All data for this study was acquired using an adapted reporting sheet (S1 File), which also included a diagram of a marked pitch (Fig 1) which allowed for accurate description of where on the pitch injuries occurred. Injuries were classed as occurring inside or between the 23-meter lines (inside: areas 1–3 and 10–12; between: areas 4–9) on the pitch. Additional data related to whether medical attention was required and if players returned to play was also collected, but due to practical difficulties in following this up and hence potential inaccuracies, this data was not included in analysis.

Sheets were completed by a combination of the authors and officials. To establish between-rater reliability, sheets were completed by the first author and the officials for the same four matches and results compared. Accuracy of reporting was further verified by the first author using video footage from one match on the last day.

Fully anonymized data was used for analysis. The number of injuries reported was calculated as total number of injuries, number of injuries per match (all were equal in length), and to allow comparison to previous literature from other sports as number of injuries per 1000 player match hours. A total of 20 matches were played in each competition, with 12 pool matches (two groups of four teams, played on a round robin basis) and 8 final matches played to determine overall competition placing. All teams in each competition comprised of 11 players on the pitch at any given time, and total player-match hours (PMH) was calculated as number of players on both teams (22 players) times total number of matches (20 in total, 12 in pools, 8 in finals) times match duration (70 minutes) divided by 60 minutes. Total player match hours were hence 513 hours per competition, divided into 308 PMH for the pool stages, and 205 PMH for the finals stages. Injury incidence was subsequently expressed as both injuries per match and injuries per 1,000 player match hours (injuries/1000 PMH) [23].

Following written informed consent, a representative from eleven of the participating countries medical personnel (five from the boys’ competition and six from the girls’) completed a questionnaire during the tournament. This involved completing an anonymized table listing all protective equipment used by their team. A sub-analysis of whether facial protective equipment used by the defending team was also completed by the researchers during 180 penalty corners (74 boys, 106 girls).

## Results

Inter-rater reliability using this study’s definition of injury was excellent with the same number, mechanism and nature of injury reported by all researchers. A breakdown of injuries in each competition during pools and finals stages, and overall totals, is presented in Table 1. Overall injury incidence was 2.20 (G) and 1.35 (B) injuries/match, which equated to 86 and 53 injuries/1000 PMH respectively. The total number and incidence of serious injuries was similar for both boys’ and girls’ competitions, but girls were over three times more likely than boys to have a minor injury overall.

**Table 1:** Total number of injuries, injuries per match, and injuries per 1000 player match hours (PMH) in boys’ and girls’ competitions.

		Boys			Girls		
		Number of injuries	Injuries per match	Injuries per 1000 PMH	Number of injuries	Injuries per match	Injuries per 1000 PMH
Pools	No time stoppage	4	0.33	13	17	1.42	55
	Time stoppage	8	0.66	26	10	0.83	32
	Total injuries	12	1.00	39	27	2.25	88
Finals	No time stoppage	3	0.38	15	8	1.00	39
	Time stoppage	12	1.50	58	9	1.13	44
	Total injuries	15	1.90	73	17	1.42	83
Overall	No time stoppage	7	0.35	14	25	1.25	49
	Time stoppage	20	1.00	39	19	0.95	37
	Total injuries	27	1.35	53	44	2.20	86

In the boys’ competition, similar incidence of minor injuries was observed in the pool and finals stages, but incidence of serious injury more than doubled between pools and finals stages from 26 to 58 injuries per 1000 PMH. Overall, boys were almost three times more likely to have a serious injury requiring a time stoppage compared to a more minor injury (14 vs. 39 injuries 1000 PMH). Girls were equally likely to be injured in pools and finals stages (88 compared to 83 injuries/1000 PMH), with a decreased incidence of minor injury as they progressed from pool to final stages (55 to 39 injuries/1000 PMH), and increased incidence of a more serious injury requiring time stoppage (32 to 44 injuries/1000 PMH). Girls were over four times more likely to have a minor injury in the pools than boys (55 vs. 13 injuries/1000 PMH), but incidence of serious injury was similar. In the finals, girls were 2.7 times more likely than boys to have a more minor injury, but boys were 32% more likely than girls to have a serious injury. This increased number of minor injuries in girls resulted in an overall increased injury incidence (86 vs. 53 injuries/1000 PMH).

The lowest number of injuries were observed in the first quarter. In the girls’ competition, number of injuries remained consistent for the second, third and fourth quarters (between 23 and 32%), but in the boys a decrease in the third (19%) from the second (33%), and slightly elevated level of injury in the fourth compared to the second (44%), was observed. The most common injury locations for boys were the torso (8 injuries, 30%), foot/ankle (6 injuries, 22%) and upper limb (5 injuries, 19%); girls were most likely to receive an injury to the foot/ankle (12 injuries, 27%), head/face (7 injuries, 16%) and the upper limb (7 injuries, 16%). The most common mechanism for both sexes was being struck by a ball or stick (44% for boys, 61% for girls), but a similar amount of injuries occurred in boys due to colliding with another player (41%, Table 2).

**Table 2:** Absolute and relative number of injuries in boys’ and girls’ competition: timing, location on body, and mechanism of injury.

		Boys						Girls					
		No time stoppage		Time stoppage		Total		No time stoppage		Time stoppage		Total	
		Number of injuries	Percentage	Number of injuries	Percentage	Number of injuries	Percentage	Number of injuries	Percentage	Number of injuries	Percentage	Number of injuries	Percentage
**Timing**	**Q1**	0	0	1	5	1	4	6	24	1	5	7	16
	**Q2**	2	29	7	35	9	33	3	12	7	37	10	23
	**Q3**	1	14	4	20	5	19	9	36	5	26	14	32
	**Q4**	4	57	8	40	12	44	7	28	6	32	13	30
**Location**	**Foot/ankle**	2	29	4	20	6	22	8	32	4	21	12	27
	**Lower leg**	1	14	0	0	1	4	4	16	2	11	6	14
	**Knee**	0	0	2	10	2	7	2	8	1	5	3	7
	**Thigh**	2	29	1	5	3	11	0	0	3	16	3	7
	**Torso**	0	0	8	40	8	30	3	12	3	16	6	14
	**Upper limb**	2	29	3	15	5	19	6	24	1	5	7	16
	**Head/face**	0	0	2	10	2	7	2	8	5	26	7	16
**Mechanism**	**Hit by ball/stick**	1	14	11	55	12	44	17	68	10	53	27	61
	**Collision with another player**	3	43	8	40	11	41	2	8	5	26	7	16
	**Trip/sprain/cramp**	3	43	1	5	4	15	6	24	4	21	10	23

A total of 20 (B) and 23 (G) injuries in total occurred within the 23-meter line regions, with 7 and 21 occurring elsewhere on the pitch. For boys, 75% of injuries occurring inside the 23-meter lines and 43% of those occurring elsewhere required time to be stopped. Severity in the girls’ competition was much lower, with 22% and 19% of injuries within the 23-meter line and elsewhere, respectively, requiring time stoppage.

Injuries occurring during penalty corners were low, with three injuries in the boys’ competition (all struck by ball, resulting in two lower limb contusions and one fractured finger) and four in the girls (struck by ball, contusions to thigh and hand). For the 180 penalty corners observed, girls were more likely to wear facial protection than boys (86.4% vs. 65.9%). 69% of participating teams returned a post-competition survey of protective equipment use, with all players reported as wearing full player protection during play (leg and mouth protection).

## Discussion

This study has presented one of the first datasets of injuries of U18 youth field hockey players during a major tournament. Differences in injuries between boys and girls were observed, with girls more likely to be injured in general than boys, mainly due to an increased number of minor injuries not requiring a time stoppage. Differences with stage of competition were observed: while injury incidence stayed similar between stages for girls, it almost doubled between stages for boys. A high number of injuries due to being struck by the ball or stick, and evidence of lack of compliance with wearing protective facial equipment during penalty corner set pieces, highlights the importance of design and use of appropriate protective equipment and correct coaching for youth athletes.

When the same method of recording injury was used, injury incidence in this study (0.95 (G) and 1.00 (B) injuries/match, 37 (G) and 39 (B) injuries/1000 PMH) differed from the 0.7 and 1.2 injuries/match and 29.1 and 48.3 injuries/1000 PMH reported for women’s and men’s adult tournaments by Theilen et al. [18]. With the inclusion of minor injuries, these values increased to 2.2 and 1.35 injuries/match (86 and 53 injuries/1000 PMH); the value for boys is similar to that of the adult game. When compared to other youth sport, these values are much higher than the 26.7 injuries per 1000 PMH reported by Freitag et al. [24] for any injury regardless of medical attention sought or time-loss, in under 21 year old rugby players, which is a broader remit than the most common definition of injury [23]. They are, however, within the 27.5 to 129.8 injuries/1000 PMH range reported in a systematic review by Bleakley et al. on injuries in adolescent rugby [25]. Injury incidence in the girls’ competition is approximately three times that of adults. This is probably due to the inclusion of multiple minor injuries as when the same reporting protocol was used, i.e., when only noting injuries requiring time stoppage, incidence was more similar. Serious injury incidence more than doubled between the pool and finals stages of the boys’ competition (0.67 to 1.50 injuries/match, 26 to 58 injuries/1000 PMH) which is not unexpected with the increased importance of match outcomes. With this increase in importance, players are more likely to take chances and place themselves in more precarious positions, which can potentially lead to injuries. Overall injury incidence in females remained the same throughout the tournament (2.25 and 2.13 injuries/match, 88 and 83 injuries/1000 PMH), but severity of injury varied: while minor injury incidence slightly decreased between pool and final stages, serious injury incidence slightly increased.

Unsurprisingly, a high number of injuries occurred at the foot and ankle (6 in boys–second most common, 12 in girls–most common) which is expected due to the nature of the sport and the height which the ball must be played at. Although technically a non-contact sport, players frequently collide with another as evidenced by the number of torso injuries (8 in boys, all requiring time stoppage; 6 in girls, 50% requiring time stoppage) where players collided with one another. Injuries in the upper limb were primarily to the hand and fingers, due to how the stick is held and how a player attempts to challenge an opponent for the ball (e.g. reaching around players and touching the ground). Of note, similar to previous findings [13, 26, 27], girls were more likely to acquire a head or face injury; in this instance, 3.5 times as many head and face injuries were observed in the girls compared to the boys, and these injuries in particular tended to require a time stoppage (100% in the boys’ competition, 71% in the girls’). A sex difference in the nature of pediatric injuries has been previously reported [28], which may be due to any number of mechanical, technical, or physiological differences. From a practical perspective, it is possible this is due to different emphasis in coaching or simply different technical capabilities. Athlete development models may hence have to consider this when deciding upon expected technical competencies as an athlete progresses.

The highest number of injuries occurred due to being hit by a ball or stick which is comparable to trends observed in adults [18, 26], and supports the importance of wearing correctly fitting protective equipment during participation. Girls were over twice as likely than boys (27 injuries vs. 12) to be injured due to being struck by a ball or stick, which highlights the importance of correct coaching of technical skills such as challenging for the ball to minimize likelihood of an injury occurring due to incorrect positioning relative to an attacking player. Coaching emphasis may be effective in reducing risk of injury during these events and may need to be considered for specific inclusion in future coaching models.

In most sports, there is an increased risk of injury occurring as a match progresses and players become more fatigued. Hockey is unusual in that it operates a ‘rolling substitute’ system, whereby players are continuously rotated into and out of play. There is hence less cumulative fatigue to lead to a sudden spike in injury rates in the third and fourth quarters of a match. Results show the lowest number of injuries in the first quarter (4 and 16% for B and G respectively), and comparable numbers in the subsequent quarters. There is typically a high incidence of strain injuries in the latter stages of team sport matches [29, 30] but this is not observed here or in adult field hockey. This may be due to overall lower cumulative fatigue. The use of rolling substitutions, and hence, lower cumulative fatigue, ensures there is no need for a player to remain on the pitch unnecessarily, and a player with a minor injury can also remove themselves from play before an injury of this kind occurs.

Most injuries in the boys’ competition occurred within the 23-meter region (20 compared to 7), in contrast to the girls’ competition where injuries were more evenly distributed across the pitch. The high number of injuries in such a small space (many of which required time stoppage) is not surprising, due to the high density of players in a small space when the ball approaches and enters the circle. As goals in field hockey must be scored from inside the circle, the intensity of play is visibly increased due to the potential significant outcomes. This intensity may explain the severity of the injuries observed particularly in the boys. Percentage of injuries observed for boys are comparable to those observed by Theilen et al. [18], but the percentage of injuries reported here for the girls is much lower. Tactically, male players are more likely to strike directly for goal at speed, whereas females often play to gain a penalty corner, often with their heads down. have their heads down. It is possible that at U18 level, these players have not yet developed similar technical and tactical skills as senior players to maximize opportunities in this region of the pitch, and instead are more likely to have a more minor incident such as being hit in a finger. These different playing styles may help to explain the rates of injury and injury severity observed in this part of the pitch.

Number of injuries occurring during the penalty corner were low, and similar to that observed in adult field hockey. These injuries were primarily contusions to the lower limb rather than due to balls being raised towards head and face level. Only one country in attendance at the competition wore specific knee protection during this set-play, and it is unclear why. Awareness of the high proportion of injuries that occur due to this mechanism may potentially encourage more teams to utilize them. While still an ongoing debate, these results suggest the penalty corner is a relatively safe set-play, despite concerns over ball speed as it enters the circle. These results do not show it is completely safe, and there are obvious risks with a ball travelling at high speeds particularly when player skill level may be lower and hence incidents are more likely to happen. This study does however show that there are potential mechanisms available for reducing the risk of an injury occurring in this area.

While team personnel reported all players wore both mouth and leg protection, unfortunately one player suffered a mouth injury without a gum shield in place. While traumatic oral injuries in field hockey are very rare, at 0.06 injuries per 1000 athlete exposures [26], there are significant consequences when they occur. Umpires hence have an important role to play in monitoring player compliance to wearing important protective equipment. Current FIH rules do not enforce wearing protective equipment; instead, players are advised strongly to wear it. Boys were less likely than girls to wear facial protection during the penalty corner (65.9% vs. 85.4%), and the reason for this is unclear. It is possible players believe use of this equipment may reduce their vision or reaction, and with some designs, this may be a genuine concern. Improved design of protective equipment to maximize vision and reduce potential issues with masks fogging up, completion and dissemination of research to investigate the effect their use has on performance, and highlighting positive role models at senior level who wear facial protection may all help to increase compliance. The importance of non-obtrusive, correctly designed and fitted protective equipment cannot be over-emphasized, as issues in these factors may in fact increase the probability of injury occurring [31]. Currently, boys and girls use the same equipment designs. Mouth protection is the most likely piece of protective equipment to be custom-made to the individual, and it is possible that this is also an issue due to observed differences in anthropometrics.

Existing injury reporting protocols do not account for all injuries that occur in field hockey, as shown by the different calculated injury rates based on the two injury definitions. The additional reporting protocol used demonstrated excellent between-rater reliability and has shown minor injuries occur throughout a tournament competition which, to now, have been unreported. It must be noted the existing protocol does capture the major injuries of a game, and additional injuries reported tend to be less serious in nature and more likely to be considered an inherent part of sporting activity. The generic classification of injuries as serious and minor may have some inaccuracies where serious injuries are not noticed and a player removes themselves from the pitch, and indeed, time may be stopped for minor injuries which do not seriously affect the athlete, but for the purposes of this study these classifications were deemed broadly acceptable. To develop the optimal injury prevention strategy in youth field hockey requires understanding the incidence and severity of all injuries. Many sports tournaments at youth level are organized by volunteer organizers and officials, and while informative, the practicality of incorporating more detailed, compulsory injury analysis requires consideration as few events have a significant body of volunteers to assist with completion of additional documentation. Provision of a clear definition as to what constitutes an injury is necessary. In addition, as is common to many sports there is no simple easy way to identify more complex yet less obvious injuries such as concussion [32] and hence include them in reporting. There may be underreporting of this injury in competition due to difficulty in diagnosis, which is something that must be addressed by sporting bodies in general.

## Conclusions

This is the first study to report injury incidence in elite U18 field hockey players in competition. By utilizing a novel, reliable approach to recording injuries in the game based upon a combination of existing FIH standard reporting and additional monitoring by tournament officials and researchers of less obvious but still impactful injuries on the pitch, this study has presented the first injury incidence data for this cohort playing the modern field hockey game. It is an essential first step in establishing the scale of, and nature of, injuries in U18 field hockey players–a critical dataset which the International Olympic Committee has specifically highlighted as lacking in this sport. Using existing protocols, injury incidence in elite U18 field hockey differs from that observed in the adult game, highlighting the importance of establishing injury incidence in the population of interest. Although the existing method of reporting captures most serious injuries, it under-reports minor injuries sustained and is an issue the international governing body needs to address. Contusions to the lower limb account for most injuries, but there is a high number of head/face injuries, particularly in females, which require further investigation as well as reduced compliance with use of facial protection in boys. These findings highlight the need for acquisition of injury data to inform and improve coaching and sports medicine practice, and sports engineering design.

## Supporting information

S1 File Reporting sheet used at tournament. This sheet was used by the authors and tournament officials for recording details related to injuries acquired by players at the 2016 U18 European Hockey Championships I.
(PDF)
