# Supplementary material for: Injury incidence in elite youth field hockey players at the 2016 European Championships
Source: PLoS One. 2018 Aug 23;13(8):e0201834. doi: 10.1371/journal.pone.0201834 (PMC6107116; doi:10.1371/journal.pone.0201834)
Supplement: S1 File — This sheet was used by the authors and tournament officials for recording details related to injuries acquired by players at the 2016 U18 European Hockey Championships I. (PDF) [file pone.0201834.s001.pdf]

Loughborough University

GOETHE UNIVERSITÄT FRANKFURT AM MAIN

Date: Time:

Competition: BOYS/GIRLS Pitch: GARRYDUFF/MARDYKE

Teams:

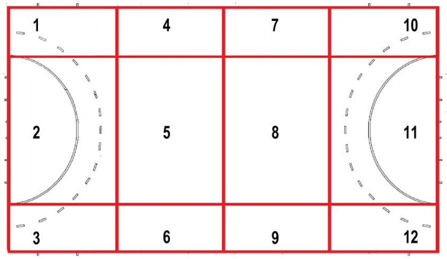

Time	Team and player number	Area of body affected	Injury type	Cause of injury	Location on pitch (code above)	Medical attention (Y/N)	Time stoppage (Y/N)	Return to play (Y/N)
